# The Evolution of Erythrocytes Becoming Red in Respect to Fluorescence

**DOI:** 10.3389/fphys.2019.00753

**Published:** 2019-06-19

**Authors:** Laura Hertz, Sandra Ruppenthal, Greta Simionato, Stephan Quint, Alexander Kihm, Asena Abay, Polina Petkova-Kirova, Ulrich Boehm, Petra Weissgerber, Christian Wagner, Matthias W. Laschke, Lars Kaestner

**Affiliations:** ^1^Institute for Molecular and Cell Biology, Saarland University, Homburg, Germany; ^2^Theoretical Medicine and Biosciences, Saarland University, Homburg, Germany; ^3^Experimental Physics, Saarland University, Saarbrücken, Germany; ^4^Center for Molecular Signaling (PZMS), Institute for Pharmacology, Saarland University, Homburg, Germany; ^5^Physics and Materials Science Research Unit, University of Luxembourg, Luxembourg City, Luxembourg; ^6^Institute for Clinical and Experimental Surgery, Saarland University, Homburg, Germany

**Keywords:** mouse model, transfusion, fluorescent protein, intravital microscopy, imaging

## Abstract

Very young red blood cells, namely reticulocytes, can be quite easily recognized and labeled by cluster of differentiation antibodies (CD71, transferrin receptor) or by staining remnant RNA with thiazol orange. In contrast, age specific erythrocyte labeling is more difficult in later periods of their life time. While erythrocytes contain band 4.1 protein, a molecular clock, so far it has not been possible to read this clock on individual cells. One concept to track erythrocytes during their life time is to mark them when they are young, either directly *in vivo* or *ex vivo* followed by a transfusion. Several methods like biotinylation, use of isotopes or fluorescent labeling have proved to be useful experimental approaches but also have several inherent disadvantages. Genetic engineering of mice provides additional options to express fluorescent proteins in erythrocytes. To allow co-staining with popular green fluorescent dyes like Fluo-4 or other fluorescein-based dyes, we bred a mouse line expressing a tandem red fluorescent protein (tdRFP). Within this *Brief Research Report*, we provide the initial characterisation of this mouse line and show application examples ranging from transfusion experiments and intravital microscopy to multicolour flow cytometry and confocal imaging. We provide a versatile new tool for erythrocyte research and discuss a range of experimental opportunities to study membrane processes and other aspects of erythrocyte development and aging with help of these animals.

## Introduction

We face many scenarios in erythrocyte research where we need to label erythrocytes. This might be in the context of determining a particular cell shape (Quint et al., 2017), transfusion experiments (Dholakia et al., 2015) or following cell age (Wang et al., 2013). There are numerous strategies available to label erythrocytes based on small molecular dyes (Haugland, 2002), antibodies (Pepe, 1968) or fluorescent proteins (Jung, 2011). Especially the latter one has particular advantages such as high biocompatibility due to cell internal translation, permanent expression and specific subcellular localization (Kaestner et al., 2014). Fluorescent proteins have proved to be useful tools to label cells (Stearns, 1995), to follow protein function (Lipp et al., 2014) or to construct biosensors (Kaestner et al., 2018). Most transgenic approaches in mammals were performed in mice making them the currently most widely used animal model. Considering the wide range of colors of fluorescent proteins (Shaner et al., 2004) and the great variety of available promoters (Chen et al., 2011), little attention has been paid to erythrocytes. Several attempts have been made to generate mice with ubiquitous expression of fluorescent proteins, e.g., Fink et al. (2010) but to our best knowledge, this has not yet led to efficient fluorescent erythrocyte labeling. However, there are several studies of primitive erythroid cells with a green fluorescent protein (GFP) fused to the ε-globin, e.g., Lucitti and Dickinson (2006), Isern et al. (2008). The use of GFP has, especially in comparison to red fluorescent proteins, numerous disadvantages, such as spectral overlap of excitation and emission with the absorption spectrum of hemoglobin (Kaestner et al., 2006), decreased penetration depth for *in vivo* investigations (Bozhanova et al., 2018) or spectral overlap with some of the most popular fluorescent biosensors, such as Ca^2+^ indicators (Lipp and Kaestner, 2014). Therefore, we set out to genetically label erythrocytes with red fluorescence in mice.

## Materials and Methods

### Mice

#### Permissions

All animal experiments were performed according to the Guide for the Care and Use of Laboratory Animals published by the U.S. National Institutes of Health and approved by the local governmental animal protection committee (approval numbers 02/2015, 06/2015 and 27/2018).

#### Breeding

Mice were kept under a standard light/dark cycle with food and water *ad libitum* in a specific pathogen-free animal facility. *Rosa26-tdRFP* mice were previously described (Luche et al., 2007) and kindly provided by Hans Jörg Fehling (Ulm University, Germany). Animals with an activated *Rosa26-tdRFP* allele (*R26-tdRFP-CMV*) were generated by crossing homozygous *Rosa26-NEO-STOP-tdRFP* mice with a heterozygous ubiquitous CMV-Cre deleter strain carrying a huCMV-Cre transgene on the X-chromosome (Schwenk et al., 1995). The resulting heterozygous *R26-tdRFP-CMV* offspring was then crossed *inter se* to obtain homozygous *R26-tdRFP-CMV* mice for analysis. Homozygous *R26-tdRFP-CMV* animals were obtained at expected Mendelian frequencies and did not show any obvious phenotypic abnormalities. The homozygous *R26-tdRFP-CMV* mice were fertile and exhibited robust red fluorescence in erythrocytes.

#### Erythrocyte Mass Parameters and Indices

Analysis of the erythrocyte mass parameters and indices was performed using a fully automated hematology analyzer (VetScan HM5, Abaxis, Union City, CA, United States). Blood was collected from *R26-tdRFP-CMV* mice with homozygous RFP expression (RFP^+/+^) and RFP^–/–^ siblings.

#### Transfusions

For transfusion experiments blood was collected from wild type (C57BL/6 mice, Charles River Laboratories, Saint-Constant, QC, Canada) and *R26-tdRFP-CMV* mice by puncture of the heart (final bleeding after 1.5% isoflurane inhalation anesthesia). Wild type erythrocytes were stained using the membrane dye PKH67 (Sigma-Aldrich, St. Louis, MO, United States). Cells were washed three times in 0.9% NaCl solution and incubated for 5 min at room temperature under rotation with PKH67 (1:200 dilution). Quenching of remaining dye was done by addition of 2% bovine serum albumin (BSA) in phosphate buffered solution (PBS) and the cells were washed again three times in 0.9% NaCl solution.

Stained wild type erythrocytes and erythrocytes from *R26-tdRFP-CMV* mice were mixed and a volume of 200 μl was retro-orbitally injected into wild type C57BL/6 mice (Charles River Laboratories, Saint-Constant, QC, Canada). The survival rate of transfused erythrocytes was analyzed by flow cytometry for 1 month. For this purpose, 10 μl blood samples of transfused mice were collected by puncture of the tail vein. The first sample was taken within 5 min after transfusion and the measured value used for normalization of the data. Analysis of the data was done using GraphPad Prism (GraphPad, La Jolla, CA, United States).

### *In vivo* Imaging Experiments

#### Animals

*In vivo* experiments were performed in 12- to 14-week old male C57BL/6 mice with a body weight of 24–26 g. The animals were bred and housed in open cages in the conventional animal husbandry of the Institute for Clinical and Experimental Surgery (Saarland University, Germany) in a temperature-controlled environment under a 12 h/12 h light-dark cycle and had free access to drinking water and standard pellet food (Altromin, Lage, Germany).

#### Dorsal Skinfold Chamber Model

Red blood cell passage of small capillaries was analyzed in the dorsal skinfold chamber model, as previously described (Danielczok et al., 2017). For chamber implantation, mice were anaesthetized by i.p. injection of ketamine (75 mg/kg body weight; Ursotamin®; Serumwerk Bernburg, Bernburg, Germany) and xylazine (15 mg/kg body weight; Rompun®; Bayer, Leverkusen, Germany). Subsequently, two symmetrical titanium frames (Irola Industriekomponenten GmbH & Co. KG, Schonach, Germany) were implanted on the extended dorsal skinfold of the animals in a stepwise procedure, as previously described (Laschke and Menger, 2016). Within the area of the observation window, one layer of skin was completely removed in a circular area of ∼15 mm in diameter. The remaining layers (striated skin muscle, subcutaneous tissue and skin) were finally covered with a removable cover glass. To exclude alterations of the microcirculation due to the surgical intervention, the mice were allowed to recover for 48 h after implantation.

#### *In vivo* Microscopy

*In vivo* microscopic analyses were performed as previously described (Brust et al., 2014). In detail, the mice were anaesthetized and a fine polyethylene catheter (PE10, 0.28 mm internal diameter) was inserted into the *carotid artery* to apply RFP-labeled erythrocytes. Then, the animals were put in lateral decubital position on a Plexiglas pad and the dorsal skinfold chamber was attached to the microscopic stage of an upright microscope (ECLIPSE Ci-L; Nikon, Tokyo, Japan) equipped with a 40×, NA 0.8, water immersion objective and a LED array (pE300ultra, CoolLED, Andover, United Kingdom) attached to a fluorescein isothiocyanate (FITC) filterset (excitation 465–495 nm, emission 515–555 nm). Up to 0.5 ml of RFP-expressing erythrocytes were transfused immediately before the imaging experiments. The microscopic images were recorded using a CMOS video camera (Prime 95B, Photometrics, Tucson, AZ, United States) connected to a PC at an acquisition speed of 415 images per second.

#### Trajectory Analysis

The recorded video sequence was analyzed using a single particle tracking algorithm, as previously described (Bächer et al., 2018). Hereby, the intensity profile of each frame was adjusted to have both the top and bottom 1% of all pixels saturated, correcting for changes in illumination and exposure time. With the aid of a tailored MATLAB script, all spherical (round) objects were detected and interconnected among all frames by cross-correlating consecutive images. We derived the respective trajectories by combining the coordinates of all classified erythrocytes over the whole video sequence.

### Single Cell Analysis

#### Bone Marrow Preparation

For the isolation of bone marrow, two mice were sacrificed by an overdose of anesthetics. The femurs and tibias were carefully excised and bone marrow cells were obtained by flushing the bones with cold Tyrode solution containing (in mM): 35 NaCl, 5.4 KCl, 10 glucose, 1 MgCl_2_, 1.8 CaCl_2_ and 10 HEPES, pH 7.4. Cells were stained with Hoechst 33258 solution (Sigma-Aldrich, St. Louis, MO, United States) at a final concentration of 10 μg/ml and a TER-119 antibody (Biolegend, San Diego, CA, United States) at 2 μg/ml in PBS at room temperature for 20 min. Cells were washed once before imaging (see below).

#### Blood Sample Preparation

Blood samples of wild type and *R26-tdRFP-CMV* mice were collected by puncture of the tail vein. Ten μl of blood were diluted in 1 ml Tyrode solution. Erythrocytes were washed three times via centrifugation at 2,500 × *g* for 3 min. The supernatant was discarded each time and the cells resuspended in Tyrode solution.

#### Confocal Imaging

Bone marrow cells or erythrocytes were suspended in PBS, 0.1% BSA and placed between two glass slides spaced by 20 μm polystyrene beads for imaging on top of a 100× objective of an inverted microscope (Nikon ECLIPSE Ti, Tokyo, Japan). A diode or solid state laser (405, 488, and 561 nm, Nikon LU-NV Laser Unit) was used as a light source for imaging. Z-stack scanning was realized with 300 nm step of piezo motor of a confocal spinning disk (CSU-W1, Yokogawa Electric Corporation, Tokyo, Japan), scanning from top to bottom for a 20 μm *z*-range. Image sequence was acquired by recording with a digital camera (Orca-Flash4.0 Hamamatsu Photonics, Hamamatsu City, Japan). Confocal slices were processed using ImageJ (Wayne Rasband, NIH, United States). 3D-rendering was performed with Vision4D (Arivis, Rostock, Germany).

#### Flow Cytometry

Fluo-4 (Thermo Fisher Scientific, Waltham, MA, United States) loading of blood samples was done for 1 h at 37°C at a concentration of 5 μM followed by one more washing step. Flow cytometer experiments were performed using a FACSAria III (Becton Dickinson, Franklin Lakes, NJ, United States). Analysis of the data was done using FlowJo 10.4.2 (FlowJo LLC, Ashland, OR, United States).

### Statistics

For all statistical analyses the Gaussian distribution of the dataset was checked by the Kolmogorov–Smirnov test. For data with Gaussian distribution the mean value ± the standard deviation was plotted. Testing for significant differences was performed with a paired *t*-test. All graph presentations and statistical tests were performed in GraphPad Prism (GraphPad Software, La Jolla, CA, United States).

## Results and Discussion

### Generating Mice With Red Fluorescent Erythrocytes

**FIGURE 1:**
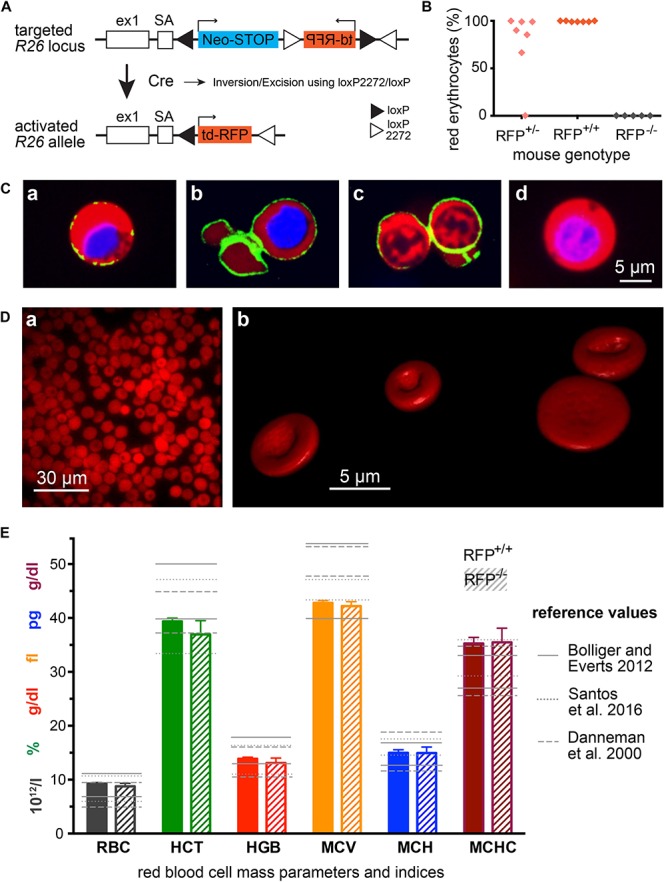
Mice with RFP erythrocytes. **(A)** Genetic strategy to generate *R26-tdRFP-CMV-Cre* mice. Mice expressing Cre recombinase under control of the cytomegalovirus (CMV) promoter were bred to *R26-tdRFP* mice carrying a *loxP*-flanked *tdRFP* gene in the *ROSA26* locus. *R26-tdRFP-CMV-Cre* mice ubiquitously express tdRFP controlled by the *ROSA26* promoter. **(B)** Percentage of red fluorescent erythrocytes in RFP^+/+^ (homozygous), RFP^+/–^ (heterozygous) and RFP^–/–^ (negative) mice. Each point represents an individual mouse. **(C)** Confocal images of cells from the bone marrow of *R26-tdRFP-CMV-Cre* mice. Beside the RFP fluorescence, the nuclei were stained with Hoechst dye (depicted in blue). Additionally a Ter119-FITC antibody was used to visualize the erythropoietic cells, **(Ca)** proerythroblast, **(Cb)** erythroblast, and **(Cc)** reticulocyte. For comparison we display a non-erythropoietic cell, and **(Cd)** from the bone marrow. **(D)** Images of RFP erythrocytes, **(Da)** epi-fluorescence microscopy of a population of erythrocytes, and **(Db)** 3D-rendered individual erythrocytes out of a z-stack of confocal images. **(E)** Erythrocyte mass parameters and indices. Displayed are the parameters erythrocyte count (RBC), haematocrit (HCT), haemoglobin (HGB), mean cellular volume (MCV), mean cellular haemoglobin (MCH), and mean cellular haemoglobin concentration (MCHC). The columns depict mean values and error bars represent the standard deviation out of 5 RFP^+/+^ and 5 RFP^–/–^ mice. The reference values are taken from Danneman et al. (2000), Bollinger and Everds (2012), and Santos et al. (2016).

To generate a mouse model with fluorescently labeled erythrocytes, we aimed for three major properties: (i) the fluorescent protein should emit in the red spectral range for reasons already outlined in the introduction; (ii) the fluorescent protein should be strongly expressed as mature erythrocytes are lacking a transcriptional and translational machinery; and (iii) the fluorescent protein should be cytosolic in order to leave the cell membrane undisturbed. *R26-tdRFP* mice encode the untargeted (and therefore cytosolic) red fluorescent protein (RFP) tandem construct tdimer2(12) (Campbell et al., 2002) under control of the *ROSA26* locus and therefore seems to fulfil all defined criteria. To activate RFP expression in erythrocytes, *R26-tdRFP* mice were crossed with a CMV-Cre line (Schwenk et al., 1995), leading to ubiquitous Cre recombinase – and thus RFP – expression. The breeding scheme is depicted in Figure 1A. Heterozygous mice from the F1 generation were crossed *inter se* to generate *R26-tdRFP-CMV* mice with a homozygous expression of RFP. While homozygous *R26-tdRFP-CMV* pups showed 100% red fluorescent erythrocytes, heterozygous *R26-tdRFP-CMV* mice expressed a rather inhomogeneous pattern (Figure 1B) and were therefore not used for further investigations. RFP-negative control mice did not display red fluorescence. The tdRFP is ubiquiously expressed in all organs as exemplified in Supplementary Figure S1. Compared to (Luche et al., 2007), which describes the initial generation and characterization of the *Rosa26-tdRFP* mouse, our results revealed bright fluorescence in erythrocytes isolated from homozygous *R26-tdRFP-CMV* mice (Figure 1C).

### Properties of Mice With Red Fluorescent Erythrocytes

We did not detect any obvious phenotypic differences in *R26-tdRFP-CMV* mice, except for the red fluorescence, when compared to wild type C57BL/6 mice. We imaged erythropoietic precursor cells from the bone marrow as outlined in Figure 1C. The translational more active cells that still contain a nucleus (Figures 1Ca,b) show a stronger expression of tdRFP compared to reticulocytes (Figure 1Cc). However, the erythrocytes showed a homogeneous red fluorescence as exemplified in Figure 1Da and the fluorescent protein was expressed at levels high enough to allow for confocal imaging of *z*-stacks and consecutive 3D-redering as shown in Figure 1Db (see also Supplementary Video 1), demonstrating that RFP expression is sufficient for fluorescence-based cell shape analysis.

Since we designed the mouse line for erythrocyte research, we next analyzed erythrocyte mass parameters and indices and plotted them in Figure 1E in comparison with text book values for laboratory mice in general (Danneman et al., 2000; Bollinger and Everds, 2012) as well as for a particular investigation on C57BL/6 mice (Santos et al., 2016). Except for MCHC all values were within the reference range. However, since MCV is on the lower end of the range and haemoglobin concentration (HGB) rather high, it is not surprising that MCHC is at the upper range or slightly above. The reason is that in the used device (VetScan HM5) the MCHC is calculated out of the MCV and the HGB using the following formula: *MCHC* = *HGB*/*n × MCV* (VetScan HM5, 2018), with *n* being the number of RBCs per volume. HGB is measured photometrically at 540 nm and MCV is the average volume of RBCs derived from the RBC histogram. Considering the differences even in the ranges in-between textbooks, we conclude that erythrocytes of *R26-tdRFP-CMV* mice are within the physiological variation. Importantly, erythrocytes from RFP^+/+^ mice were not different when compared to those of RFP^–/–^ mice demonstrating the RFP itself does not influence the integrity of the parameters’ measurement procedure/principle.

### Transfusion Experiments With Red Fluorescent Erythrocytes

**FIGURE 2:**
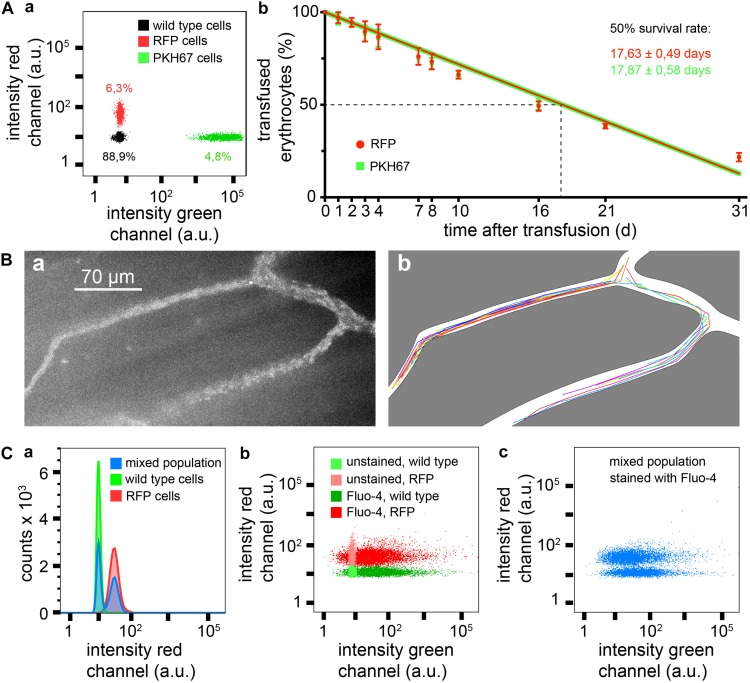
Measurements based on RFP erythrocytes. **(A)** Survival of transfused erythrocytes, **(Aa)** flow cytometric sample measurement immediately after transfusion showing 6.3% of RFP cells and 4.8% of PKH67 stained cells, and **(Ab)** statistical analysis of four transfusions over a time period of 1 month, RFP and PKH67 stained erythrocytes were simultaneously transfused in the same mouse to face identical conditions. **(B)** Intravital imaging of transfused RBCs, **(Ba)** maximal intensity projection of a time series of images, and **(Bb)** trajectories of individual erythrocytes from the same image stack as shown in Ba. **(C)** Flow cytometry of Fluo-4 loaded erythrocytes from RFP^+/+^ and RFP^–/–^ mice, **(Ca)** histograms of red fluorescence of RFP^–/–^ and RFP^+/+^ erythrocytes and of a mixed population (simulating transfusion), **(Cb)** dot plot for red vs. green fluorescence of Fluo-4 stained and unstained RFP^+/+^ and RFP^–/–^ erythrocytes, and **(Cc)** dot plot for red *vs.* green fluorescence of a Fluo-4 stained mixed population of RFP and wild type erythrocytes (simulating transfusion).

To test the transfusion ability of erythrocytes isolated from *R26-tdRFP-CMV* mice we wanted to compare the survival of these cells after transfusion with cells fluorescently labeled with the PKH67 dye (green fluorescence), a marker that proved to be successful in previous transfusion experiments in mice (Wang et al., 2013). To enable a direct comparison under identical conditions both RFP erythrocytes and PKH67 labeled erythrocytes were simultaneously transfused into the same mouse. Figure 2Aa depicts a flow cytometric analysis measurement of a blood sample after transfusion. Figures 2Ab shows the time course of 1 month of the erythrocyte survival after transfusion. There was no difference between RFP erythrocytes and PKH67 labeled ones. The half life of the transfused erythrocytes was 17.63 ± 0.49 days for RFP and 17.87 ± 0.58 days for PKH67 labeled cells. Considering that the mean erythrocyte life span varies between different mouse strains in the range of 38–42.5 days and also taking into consideration that the random destruction can vary between 0.6 and 1.3% of erythrocytes per day (Horky et al., 1978), our results are in the same range as reported in previous studies where, e.g., for the popular biotinylation a half life of 20.5 ± 2.1 days was determined (Dholakia et al., 2015).

Taken together, these data demonstrate that the RFP mice are suited as erythrocyte donors for transfusion experiments. If reticulocytosis is induced in these donor mice, the reticulocytes can easily reach counts of 30% (Wang et al., 2013). If then reticulocytes are enriched, for example by using CD71-coated magnetic beads, a pure reticulocyte preparation can be transfused and such, membrane processes or other aspects of erythrocyte development and aging can be investigated, either directly *in vivo* (see below) or by blood sampling at defined time points.

### *In vivo* Measurements of Transfused Red Fluorescent Erythrocytes

Erythrocyte properties in flow and in particular in the circulation differ from erythrocytes in stasis. This starts with cell shape (Kihm et al., 2018) but also applies to Ca^2+^ handling (Danielczok et al., 2017) and other parameters. To investigate erythrocytes *in vivo*, the model of the dorsal skinfold chamber has proven to be a useful tool (Menger et al., 2002). Figure 2B provides a proof of principle experiment demonstrating that the fluorescence intensity of the RFP cells is sufficient for this kind of investigations. A single particle tracking algorithm was applied to extract the trajectories of individual cells, which can be used to investigate flow properties of erythrocytes (Bächer et al., 2018). In addition to Figure 2B we provide Supplementary Video 2 at a different magnification to illustrate the experimental opportunities.

### Flow Cytometry With Additional Fluorescent Labeled Red Fluorescent Erythrocytes

Fluorescein-based dyes are very popular for antibody labeling as well as for functional biosensors. For the special but very important purpose of measuring Ca^2+^ homeostasis in erythrocytes, the fluorescein-based Ca^2+^ indicator Fluo-4 is the only serious option (Kaestner et al., 2006). Therefore we took Fluo-4 as an example to demonstrate that RFP and Fluo-4 together work well in erythrocytes. Figure 2C shows flow cytometric measurements of wild type and RFP erythrocytes loaded with Fluo-4, in separate measurements as well as in a mixed population, demonstrating a clear differentiation of the two populations and the lack of crosstalk between Fluo-4 and RFP.

## Summary

We generated a novel mouse strain to robustly label erythrocytes with red fluorescence. We demonstrate that the RFP fluorescence intensity in erythrocytes is sufficient for a range of applications in erythrocyte research such as 3D-shape analysis (Figure 1Db and Supplementary Video 1), transfusion experiments (Figures 2A,Ba), intravital microscopy (Figure 2B and Supplementary Video 2) and fluorescence multiplexing (Figures 2Aa,C).

## Data Availability

All datasets generated for this study are included in the manuscript and/or the Supplementary Files.

## Ethics Statement

All animal experiments were performed according to the Guide for the Care and Use of Laboratory Animals published by the U.S. National Institutes of Health and approved by the local governmental animal protection committee (approval numbers 02/2015, 06/2015, and 27/2018).

## Author Contributions

LK and LH defined the study, planned the experiments, interpreted the data, and drafted the manuscript. PW, SR, and UB designed the experimental animal study. LH, ML, GS, AK, AA, PP-K, and SQ performed the data acquisition and analysis. ML, CW, PW, and UB critically revised the manuscript. All authors listed have made a substantial, direct and intellectual contribution to the work, and approved it for publication.

## Conflict of Interest Statement

The authors declare that the research was conducted in the absence of any commercial or financial relationships that could be construed as a potential conflict of interest.
