# Supplementary figures and images for: The Evolution of Erythrocytes Becoming Red in Respect to Fluorescence
Source: Front Physiol. 2019 Jun 19;10:753. doi: 10.3389/fphys.2019.00753 (PMC6593091; doi:10.3389/fphys.2019.00753)

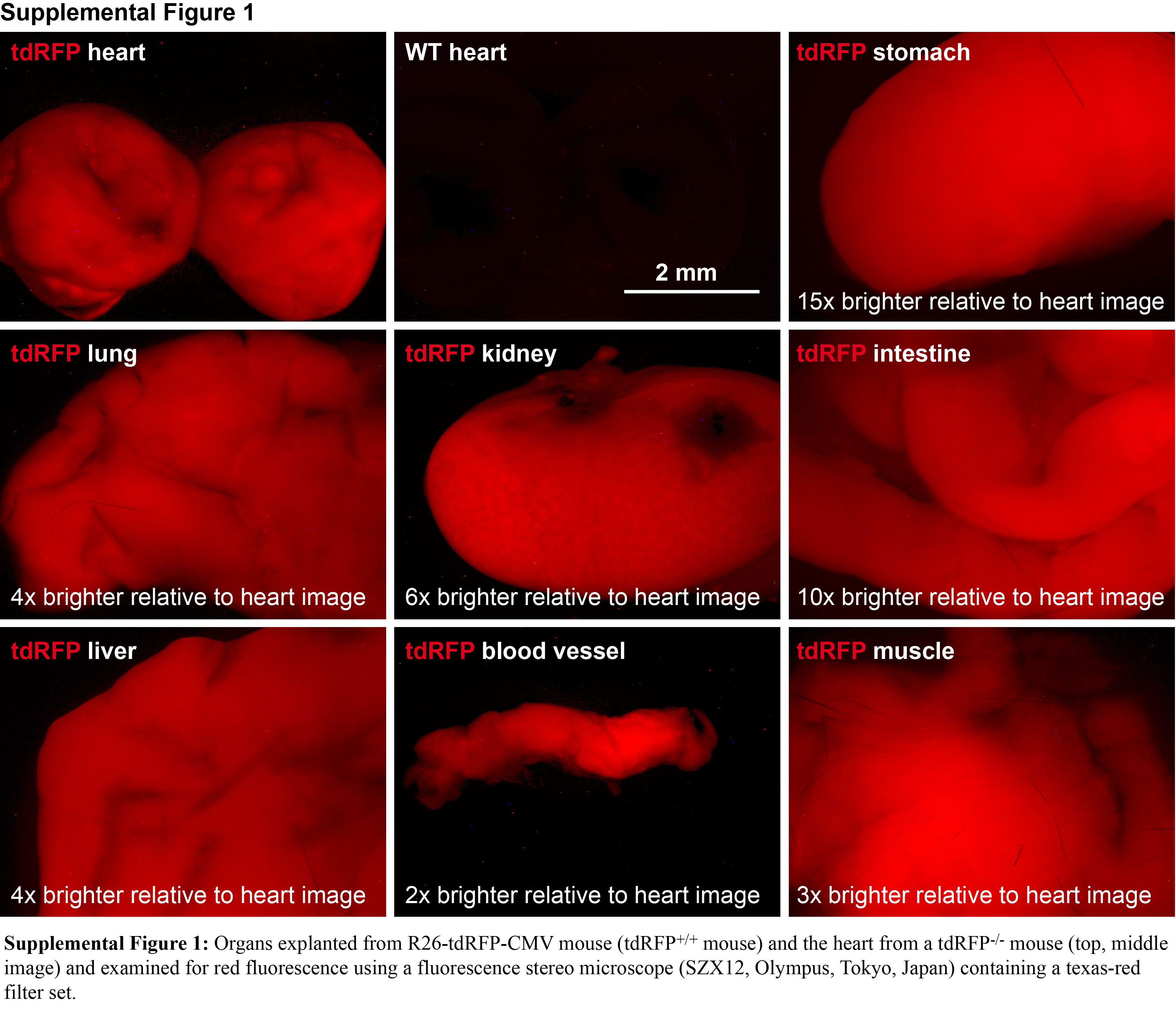

Supplement: Supplementary file 1 [file Image_1.JPEG]
